# Distribution of Bacterial Endophytes in the Non-lesion Tissues of Potato and Their Response to Potato Common Scab

**DOI:** 10.3389/fmicb.2021.616013

**Published:** 2021-02-09

**Authors:** Wencong Shi, Gaoya Su, Mingcong Li, Bing Wang, Rongshan Lin, Yutian Yang, Tao Wei, Bo Zhou, Zheng Gao

**Affiliations:** ^1^State Key Laboratory of Crop Biology, Shandong Agricultural University, Tai’an, China; ^2^College of Life Sciences, Shandong Agricultural University, Tai’an, China; ^3^National Engineering Laboratory for Efficient Utilization of Soil and Fertilizer Resources, Tai’an, China; ^4^Agriculture Research Institute of Zaozhuang, Zaozhuang, China

**Keywords:** plant microbiome, potato common scab, bacterial community distribution, endophytic source tracking, endophytes

## Abstract

The response of plant endophytes to disease within infected tissues has been well demonstrated, but the corresponding response of endophytes in non-lesion tissues remains unclear. Here, we studied the composition and distribution of bacterial endophytes in potato roots (RE), stems (SE), and tubers (TE), and explored the response of endophytes in non-lesion tissues to potato common scab (PCS), which is a soil-borne disease caused by pathogenic *Streptomyces* and results in serious losses to the global economy every year. Via high-throughput sequencing, it was seen that the composition of endophytes in roots, stems, and tubers had significant differences (*P <* 0.05) and the distribution of the bacterial communities illustrated a gradient from soil to root to tuber/stem. PCS significantly reduced bacterial endophytes α-diversity indexes, including ACE and the number of observed operational taxonomic units (OTUs), of RE without significantly reducing the indexes of SE and TE. No significant effect on the composition of endophytes were caused by PCS in roots, tubers, or stems between high PCS severity (H) and low PCS severity (L) infections at the community level, but PCS did have a substantial impact on the relative abundance of several specific endophytes. *Rhizobium* and *Sphingopyxis* were significantly enriched in root endophytes with low PCS severity (REL); *Delftia* and *Ochrobactrum* were significantly enriched in stem endophytes with low PCS severity (SEL); *Pedobacter*, *Delftia*, and *Asticcacaulis* were significantly enriched in tuber endophytes with high PCS severity (TEH). OTU62, a potential PCS pathogen in this study, was capable of colonizing potato tubers, roots, and stems with few or no symptoms present. Co-occurrence networks showed that the number of correlations to OTU62 was higher than average in these three tissue types, suggesting the importance of OTU62 in endophytic communities. This study clarified the distribution and composition of potato endophytes in tubers, roots, and stems, and demonstrated the response of endophytes in non-lesion tissues to PCS.

## Introduction

Endophytes are bacteria and fungi that exist saprophytically in plants, many are parasitic or symbiotic and live in healthy plant tissues at certain or at all life stages but, generally, do not cause damage to plant health. Endophytes are widely distributed in roots, stems, leaves, seeds, other tissues, and intercellular spaces of various plants (Hallmann et al., 1997; Stone, Bacon, and White, 2000; Berg and Hallmann, 2006). Various endophytes have coevolved with plants, generating a series of special biological functions, such as fixing nitrogen, producing IAA, and generating secondary metabolites to promote plant growth and protect against plant disease (Lyons et al., 1990; Hinton and Bacon, 1995; Baldani et al., 1997; Leuchtmann, 2006; Sun et al., 2008).

Endophytes are very diverse and vary in composition, distribution, and colonization in different species, tissues, and life stages of plants. They inhabit different ecological niches and change dynamically (Tervet and Hollis, 2002; Schulz and Boyle, 2005; Leo and Dirk, 2008; You, 2008). Some endophyte species are associated with underground tissue only while others are aboveground specific (Bulgarelli et al., 2013). Some studies have suggested that the distribution of endophytes in plants decreases gradually from bottom to top (root to leaves) and that the abundance of endophytes decreases in a similar pattern, thought to correspond to endophyte movement within the plant (Rosenblueth and Martínez-Romero, 2004). Chi et al. (2005) found that *Rhizobium* could colonize the root surface and then enter the interior of rice roots, becoming endophytic before rising to the stem, leaf sheath, and finally leaf. The diversity and abundance of endophytes in the root of reeds were higher than those of in the above-ground tissues (Rosenblueth and Martínez-Romero, 2004). These phenomena are consistent with the hypothesis that most endophytes are soil-derived while others might be derived from seeds, air, and the other plant compartments (Compant et al., 2012; Ma et al., 2013). Given the plant and tissue specificity of endophytes, it is essential to clarify their composition in specific tissues of a plant and their distribution in different tissues of the same plant.

Most endophytes inhabit healthy plants and have a variety of biological functions, such as disease control. More specifically, Ji et al. (2008) isolated and cultured endophyte *Bacillus subtilis* Lu144 from mulberry leaves and showed that it was resistant to mulberry bacterial wilt. Nejad and Johnson (2000) obtained endophytes from rapes that could inhibit the wilt of rape and tomato. Reiter et al. (2002) analyzed the structure and diversity of potato endophytes before and after infection with Blackleg based on 16S rRNA and found that the bacteria community diversity increased in infected plants. Rhizosphere microorganisms are considered to be the first line of defense against plant diseases, while endophytes are the second line of defense against plant diseases (Dini-Andreote, 2020). For example, after banana was infected with *Fusarium* wilt pathogen, the plant recruited specific antagonistic endophytes (Lian et al., 2008). The wilt fungus *Rhizoctonia solani* in infected sugar beet roots induced disease-suppressive functions in the endophytic root microbiome and recruited certain antagonistic endophytes like *Chitinophagaceae* and *Flavobacteriaceae* (Carrión et al., 2019). The diversity of endophytes increased after tobacco was infected with bacterial wilt, and the endophytes in infected plants could be traced to the rhizosphere and the surrounding soil, further supporting facultative recruitment of endophytes in the presence of disease (Reiter et al., 2002; Hu et al., 2020). These studies demonstrate the endophytic microbiota’s ability to respond to plant diseases. Many of these diseases have a common feature—they cause damage to multiple plant components simultaneously, including the roots, which can recruit microbes from the soil. Thus, we explored how endophytes in uninfected (non-lesion) tissues responded to diseases elsewhere in the plant.

According to FAOSTAT data in 2018^1^, potato was the world’s fourth largest food crop, and its production has already increased to 368 million tons, making it a significant contributor to world food supply. Despite the vital role potato has in the global food chain, few studies have explored the composition and distribution of potato endophytes and their corresponding relationship to disease. Potato common scab (PCS) is considered to be one of the largest soil-borne diseases in the potato market, causing serious economic losses around the world. PCS causes shallow and deep corky blemishes on the surface of potatoes with the phytotoxin thaxtomin (Leiminger et al., 2013; Arslan et al., 2018; Sarwar et al., 2018), adversely affecting both the storage and taste of potatoes (Getahun, 2018). The pathogen that causes PCS is mainly *Streptomyces*, and includes subtypes such as *S. scabies*, *S. acidiscabies*, *S. turgidiscabies*, *S. stelliscabiei*, and *S. bottropensis*. Some studies have reported that the occurrence of PCS may be regulated by certain microorganisms. Some microorganisms, such as *Brevibacillus*, *Bacillus*, and *Pseudomona*s, could reduce the incidence or severity of PCS (Meng et al., 2013; Arseneault et al., 2015; Chen et al., 2017). In addition to focusing on a single biocontrol strain, several studies are exploring soil microbiota regulation as a method to control PCS (Rosenzweig et al., 2012; Tomihama et al., 2016). Our previous studies also found that the geocaulosphere soil (GS) microbiome was correlated with PCS severity (Shi et al., 2019). These studies have revealed that the soil bacterial community is related to PCS, but the specific relationships between endophytes and PCS remain elusive. PCS has no definitive effects on the physiological health of potato plants other than the tuber epidermis. Little is known about whether the endophytes in non-lesion tissues respond to PCS in a concerted way or at all.

We conducted a field experiment in Jiaozhou City, Shandong Province, China in 2015 as previously described (Shi et al., 2019). Potato tubers, roots, and stems were collected to identify endophytic bacterial communities via high-throughput sequencing. According to the severity of PCS, samples were divided into two groups: high (H) and low (L) PCS severity. Here, we quantify: (i) the composition and distribution of bacterial endophytes in potato roots, stems, and tubers and (ii) the PCS response of potato endophytes in non-lesion tissues.

## Materials and Methods

### Sample Collection and Processing

A field experiment was conducted in 2015 as described in Shi et al. (2019). PCS-sensitive cultivar potatoes (Favorita 15) were cultivated in a field in Anjiatun Village of Jiaozhou City, Shandong Province, China (34.248727°N, 119.816724°E, 22.9 ma.s.l.) in 2015. The potatoes were harvested 80 days (mature plants) after planting in early November. We sampled 10 potato plants with uniform growth, no insect pests, or mechanical damage for their endophytic communities of three different tissues (root, tuber, and stem). Among the 10 plants, half had high PCS severity (H, PCS severity ≥4, specimens No. 1–5) and half had low PCS severity (L, PCS severity 1–2, specimens No. 6–10). PCS severity was quantified using methods outlined in Shi et al. (2019). PCS severity measurement were based on the percentage of surface area covered by lesions, classifying potatoes into the following nine ascending grades of severity: 0% (no scab); 0.1–0.8; 0.9–2.8; 2.9–7.9; 8.0–18.0; 18.1–34.0; 34.1–55.0; 55.1–77.0; and 77.1–100%. The soil microbiota of the soil–root system compartments of these 10 plants have been previously quantified (Shi et al., 2019). Here, the soil microbiota data of the geocaulosphere (tuber surface) soil (GS) and rhizosphere soil (RS) were reselected and analyzed together with the endophytic communities of tubers (TE), roots (RE), and stems (SE) to trace the source of endophytes. Raw sequence data of the soil communities are publicly available in the NCBI Sequence Read Archive (SRA) under the Bioproject number PRJNA477767 (Shi et al., 2019).

Sampling of each plant individual included roots, stems, tubers, and soil. The stems (10–30 cm aboveground) were cut and collected after the leaves were removed. Tubers and roots were carefully collected with aseptic stainless-steel shovels, and the soil that was loosely attached to the tubers and roots was gently removed. All samples were transported to the laboratory within 12 h under low temperature conditions (4°C). GS and RS were classified as soils tightly attached to the surface (about 1 mm) of tubers and roots, respectively. After soil sampling, the roots, stems, and tubers were washed three times with sterile, distilled water, then soaked in 70% ethanol for 2 min, followed by sterile water twice, then soaked in 3% sodium hypochlorite twice, for 1 min each, and finally washed with sterile water three times at 2 min each. We subsequently used the last sterile water rinse to inoculate a control plate to check the disinfection process. All the plant tissue was then cut into small pieces, ground into a homogenate, and stored at −80°C as preparation for DNA extraction.

### DNA Extraction

We used the E.N.Z.A.^TM^ Plant DNA Kit (Omega, United States) to extract DNA from the three kinds of potato tissue samples according to the manufacturer’s instructions. We measured the concentration and quality of DNA with a NanoDrop 2000 spectrophotometer (Thermo Fisher Scientific, United States), and all DNA samples were stored at −80°C until further analysis.

### Illumina MiSeq Sequencing and Analysis

The sequencing of endophyte communities and soil communities were carried out in a single batch with the same amplification primers, libraries construction, and sequencing conditions. The raw sequence data for the endophytic communities is publicly available under Bioproject number PRJNA657530.

The primers 341F (5′–CCTACGGGNGGCWGCAG–3′) and 805R (5′–GACTACHVGGGTATCTAATCC–3′) were used to amplify the V3–V4 hypervariable region of the 16S rRNA gene. Per the manufacturer’s instruction, amplicon libraries were constructed using the NEB Next^®^ Ultra^TM^ DNA Library Prep Kit for Illumina (NEB, United States), and index codes were added. Amplicon libraries were sequenced on a MiSeq PE250 sequencer (Illumina, United States) with 250–bp paired–end read. To optimize data quality, we used USEARCH v. 9.2 software to merge pairs, remove primers, and connectors, and filter out low-quality and short sequences. Then, we removed chimeras and generated operational taxonomic units (OTUs) at 97% similarity. We generated an OTU profile for each of our 50 samples according to the RDP database, which included about 34,621 sequences per sample. We then removed any OTUs assigned as chloroplasts or mitochondria and counted the relative abundance of taxonomic profiles at the level of phylum and genus.

### Statistical Analysis

Based on the OTU profile, we calculated α-diversity indices, including ACE, Chao1, and Obs (the number of observed OTUs) of endophytes and soil bacteria communities using the *diversity* function from the “vegan” package in R 3.6.3.

We used a repeated-measures design, where microbial sampling was performed on the same individuals but in different tissues, so we used a multivariate approach (sparse Partial Least Squares Discriminant Analysis—sPLSDA) to discern subtle differences in the bacterial community composition between different tissue types and to eliminate individual variation. We performed sPLSDA prior to rarefaction (Cao et al., 2016). sPLSDA was run using the R package “mixOmics,” with the input OTU profile normalized using log ratio transformations (lg (X + 0.0001)). Analyses of similarities (ANOSIM) was performed with the *anosim* function from the “vegan” package in R with the weighted unifrac matrix.

We traced the source of endophytes of each plant tissue sample using Fast Expectation-maximization for Microbial Source Tracking (FEAST) (Shenhav et al., 2019). All scripts were based on the main program “FEAST_scr/src.R”^2^. We used the following R packages to organize, analyze, and visualize the data: package “vegan,” “dplyr,” “doParallel,” “foreach,” “mgcv,” “reshape2,” “ggplot2,” “cowplot,” “Rcpp,” and “RcppArmadillo.” We traced RE from GS, RS, and SE, SE from GS, RS, and RE, and TE from GS, RS, SE, and RE. We expressed the sources of different samples as percentages of the total contribution, which were calculated from the average of the FEAST results per sample.

In order to explore the differences in the endophyte communities between different tissues, we used STAMP (Parks et al., 2014) to identify specific endophytes whose relative abundances were varied between the H and L group via Welch’s *t*-test with the parameters of “Two-sided.”

We used Molecular Evolutionary Genetics Analysis (MEGA7) to infer the phylogenetic affiliations and relationships of the strains using NCBI’s Microbes BLAST^3^ aligned sequence reads. A phylogenetic tree was calculated by neighbor-joining method and the results of 1,000 bootstrap trials were shown at the nods. The evolutionary distances were computed using the Jukes-Cantor method and are in the units of the number of base substitutions per site.

The co-occurrence network was constructed to assess the importance of OTU62 in the endophytic community. We calculated the Pearson’s correlation coefficient between OTUs via the “*rcorr*” function from “Hmisc” package in R, and kept the correlations of *P* < 0.05 to construct the network. The network was visualized using Gephi.

For all statistical analyses, *P* < 0.05 was considered significant.

## Results

### Diversity of Endophytes in Potatoes

**FIGURE 1:**
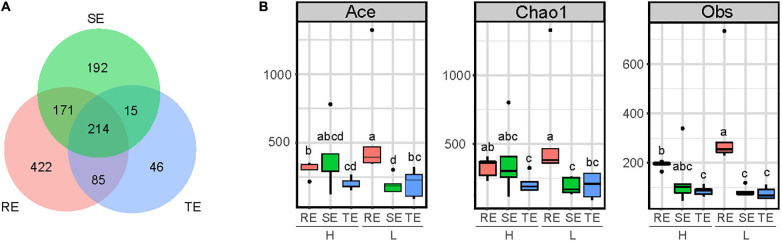
α-diversity of SE, TE, and RE. **(A)** Number of shared OTUs of RE, SE, and TE. **(B)** Ace, Chao1, and Obs indexes in RE, SE, and TE with high (H) and low (L) PCS severity. Different letters indicate significant differences at the 5% level (*P* < 0.05).

A total of 2,455 OTUs were obtained from plant endophytic (*N* = 1,202) bacterial communities (RE, SE, and TE) and soil (*N* = 2,399) bacterial communities (GS and RS). Among the endophytes from different plant tissues, there were 983 OTUs from roots, 561 OTUs from stems, and 353 OTUs from tubers. Endophytic bacteria from these three tissues types shared 214 OTUs, accounting for 21% of RE, 38% of SE, and 60% of TE (Figure 1A). From OTU profiles, α-diversity indices (Chao1, ACE, and Obs) for each endophytic bacteria community were estimated (Figure 1B). In samples with low PCS severity (L), richness indices Chao1, ACE, and Obs of roots (REL) were significantly higher than those of stems (SEL) and tubers (TEL) (two-tailed Wilcoxon test, *P* < 0.05). Meanwhile, in samples with high PCS severity (H), Chao1, ACE, and Obs indices of roots (REH) were only significantly higher than those of tubers (TEH) but not stems (SEH). PCS significantly reduced Ace and Obs indexes of RE without significantly reducing the indexes of SE and TE. There were no significant differences between SE and TE.

### Distribution of Endophytes in Potatoes

**FIGURE 2:**
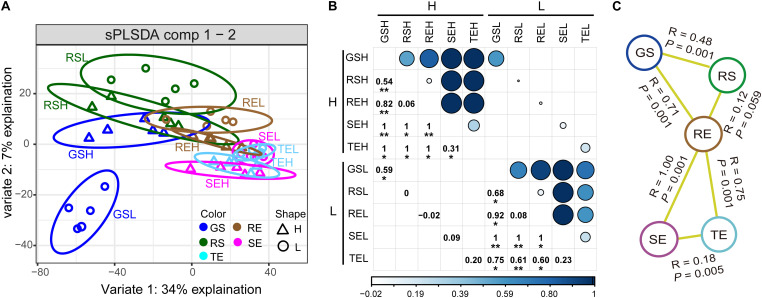
Endophytic bacterial community distribution. **(A)** sPLSDA showed a gradient distribution from soil (GS and RS) to roots (SE) to tubers and stems (TE and SE). **(B)** Visualization of differences (ANOSIM) between endophytic bacterial communities with high/low PCS severity. Numbers and dots of different sizes and colors are proportional to R value. **(C)** Significantly different (ANOSIM, *P* < 0.05) bacterial community characteristics among different tissue types. Asterisks indicates significant differences (^∗^*P* < 0.05, ^∗∗^*P* < 0.01).

To reveal the bacterial community distribution, sPLSDA and ANOSIM were performed (Figure 2). The distribution of the bacterial communities showed a gradient from soil to roots to tubers/stems (GS/RS to RE to TE/SE). In both samples with high (H) and low (L) PCS severity, there were significant differences among these three endophytic bacterial communities from different compartments (ANOSIM, *P <* 0.005). When we focused on the effect of PCS, only GS had significant differences between H and L groups, while the endophytic communities from the three types of plant tissues had no significant differences. TE had no significant differences (*R* = 0.20, *P* < 0.10) between H and L groups but showed more marginally significant differences than H and L groups in RE (*R* = 0, *P* > 0.10) and SE (*R* = 0.09, *P* > 0.10) communities. The difference between TE and RE of the L group (*R* = 0.60, *P* < 0.01) was smaller than that of the H group (*R* = 1, *P* < 0.05), indicating that PCS increases the difference between the tuber and root endophytic communities.

### The Source of Endophytes

**FIGURE 3:**
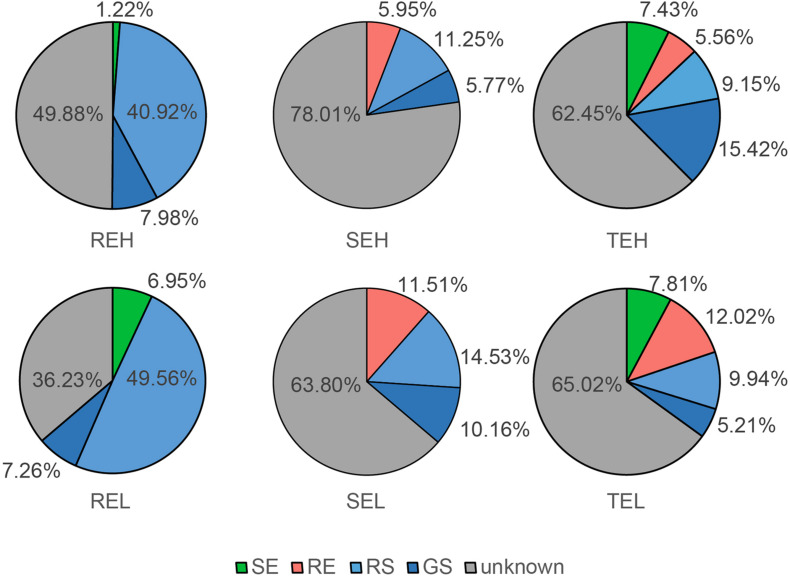
Source trace of endophytic bacterial community in potato roots, stems, and tubers.

With FEAST, we tracked the endophyte sources of roots, tubers, and stems (Figure 3). The main source of root endophytes was RS, with a small proportion of endophytes traced to GS and stem endophytes. The main sources of stem and tuber endophytes were unknown, but some (∼30%) of them were traced to GS, RS, and root communities. These results help contextualize the gradient distribution of bacterial communities in soil, roots, and tubers/stems, and their predicted origin. There were no observable differences in the source of endophytes between H and L groups of SE, TE, and RE.

### Composition of Endophytes in Potatoes

**FIGURE 4:**
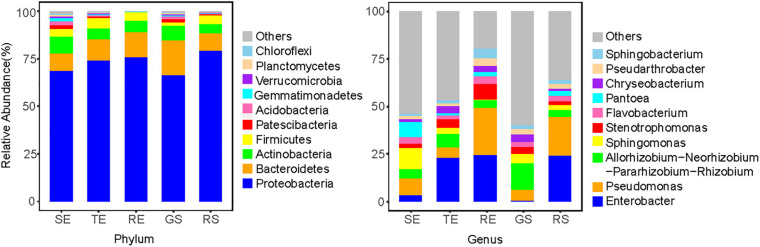
Top 10 endophytes of SE, TE, and RE at the phylum and genus level.

To further characterize the endophyte community composition among different tissues types, the top 10 endophytes at the phylum and genus levels were shown (Figure 4). In general, soil microorganisms and potato endophytes had similarities in their dominant phyla, which were *Proteobacteria*, *Bacteroidetes*, *Actinomycetes*, and *Firmicutes*. But we saw differences in the relative abundance of taxa among different plant tissues types and soil samples. For example, at the genus level, *Sphingomonas* and *Pseudomonas* were dominant bacteria in SE, *Enterobacter* and *Rhizobium* were abundant in TE, *Pseudomonas* and *Enterobacter* were abundant in RE and RS, and *Rhizobium* and *Pseudomonas* were abundant in GS. Among the five sample types, we identified 769 genera, among which 517 were identified in plant samples and 753 in soil samples, underscoring the richness of microorganisms in soil vs. endophytes on plants.

**FIGURE 5:**
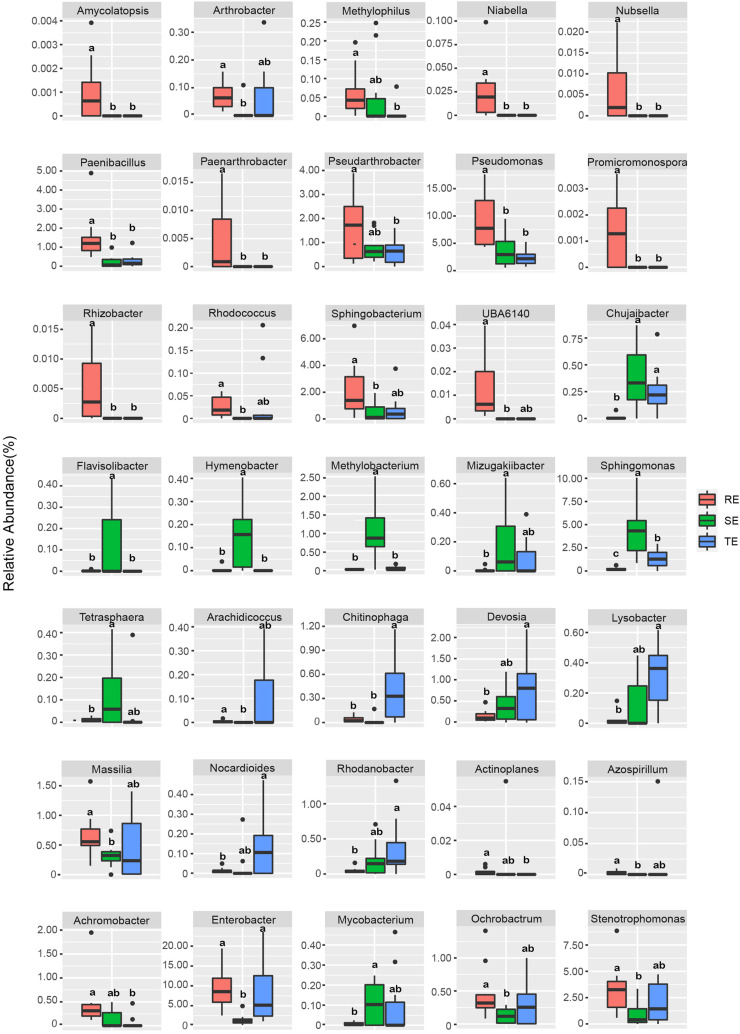
Endophytes with significant differences in RE, SE, and TE at the genus level.

We used Welch’s *t*-test to identify endophytes with significant differences between different plant tissue types. The relative abundance of 35 endophytes was significantly different in different tissues at the genus level (Figure 5). For example, *Amycolatopsis*, *Pseudomonas*, *Rhizobacter*, and *Promicromonospora* were enriched in RE, *Flavisolibacter*, *Hymenobacter*, *Methylobacterium*, and *Sphingomonas* were enriched in SE, and *Chitinophag* was enriched in TE.

**FIGURE 6:**
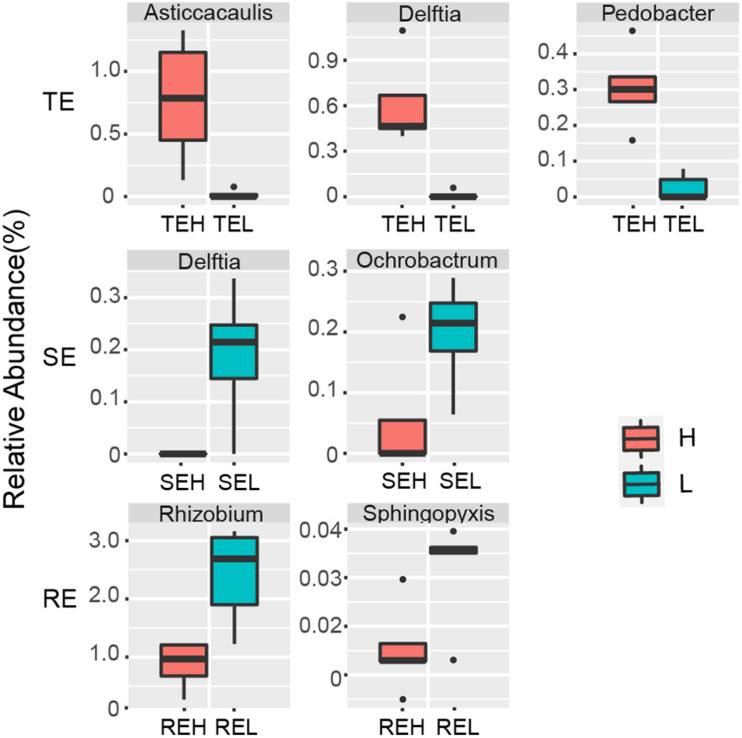
Endophytes with significant differences between group H and group L in RE, SE, and TE at the genus level.

We saw that with PCS infection the relative abundance of seven endophytes was significantly different between H and L groups. *Pedobacter*, *Delftia*, and *Asticcacaulis* were significantly enriched in tubers with high PCS severity; *Delftia* and *Ochrobactrum* were significantly enriched in stems with low PCS severity and *Rhizobium* and *Sphingopyxis* were significantly enriched in roots with low PCS severity (Figure 6).

### Potential Pathogenic *Streptomyces* in Potato Endophytic Communities

**FIGURE 7:**
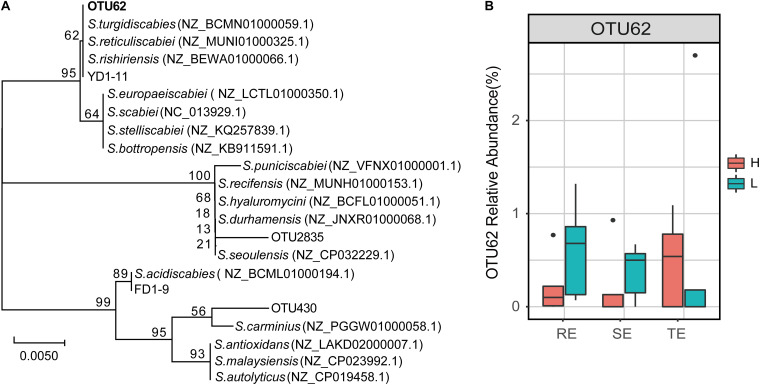
The potential PCS-causing *Streptomyces* OTU. **(A)** Phylogenetic tree for 16S rRNA gene sequences of the *Streptomyces* OTUs and pathogen strains of PCS (FD1-9 and YD1-11 are *S. acidiscabies* and *S. turgidiscabies* isolated in our previous studies, respectively). The tree was calculated using the neighbor-joining method and the results of 1,000 bootstrap trials were shown at the nodes. The evolutionary distances were computed using the Jukes-Cantor method and are in the units of the number of base substitutions per site. **(B)** Relative abundance of OTU62 in roots, stems, and tubers with high and low PCS severity.

The genus *Streptomyces* was assigned to three OTUs: OTU62, OTU430, and OTU2835. In a previous study, 12 pathogenic *Streptomyces* strains were isolated from potato peels, which were divided into *Streptomyces acidiscabies* (e.g., FD1-9) and *S. turgidiscabies* (YD1-11) from the same sample. We found that OTU62 was 100% identical to the sequence of strain YD1-11, while OTU430 and OTU2835 were not 100% identical with the any pathogenic strains (Figure 7). This illustrated that OTU62 was the potential pathogen for this study. By observing the relative abundance of OTU62, we found that it was not significantly different between H and L groups in TE, RE, and SE. Notably, OTU62 could be detected in roots, tubers, and stems, even with mild or no symptoms of PCS infection. This finding provides novel insight into the presence and spread of pathogenic *Streptomyces*.

**FIGURE 8:**
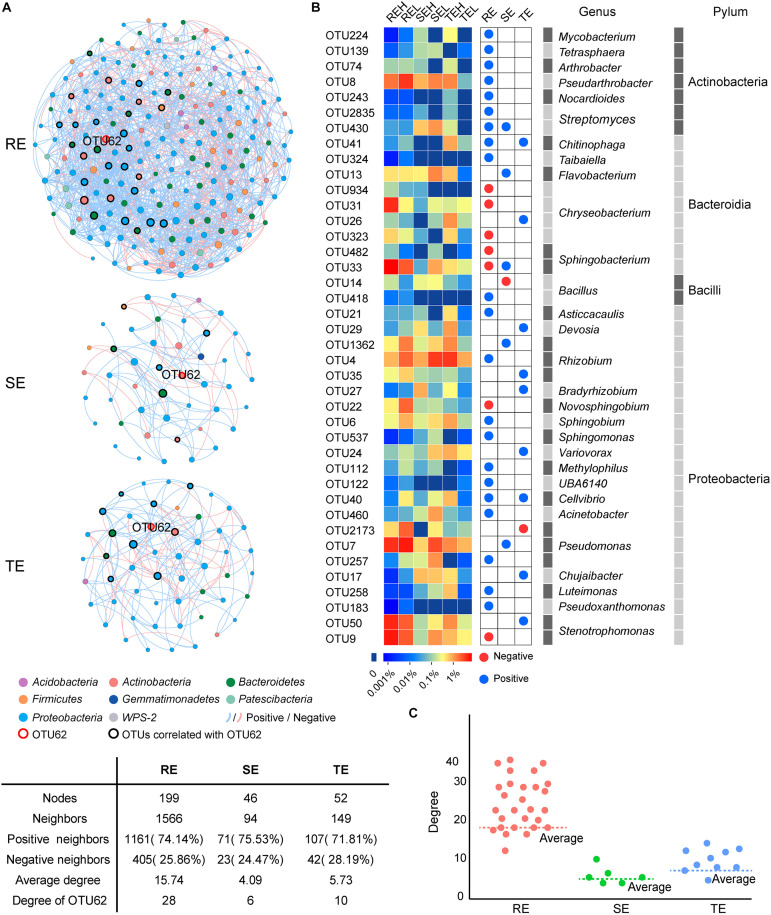
Co-occurrence networks of SE, TE, and RE at the OTU level. **(A)** The endophytic network in root, stem, and tuber. A connection represents a significant correlation (*P* < 0.05). Blue and red lines, respectively, represent the positive and negative and correlations between OTUs. The nodes in different colors represent different bacteria at phylum, and the size of each node is proportional to the degree of each OTU. **(B)** The heatmap showed the relative abundance and taxonomic information of OTUs correlated with OTU62. Blue and red nodes represent the positive and negative correlation with OTU62, respectively. **(C)** Scatter plot showed the degree of OTUs correlated with OTU62 in the three endophytic networks.

We conducted a co-occurrence network analysis to evaluate the importance of OTU62 in endophytic networks of three tissue types (Figure 8). In these three endophytic networks, a total of 40 OTUs were significantly correlated with OTU62, which mainly belonged to *Chryseobacterium* (four OTUs), *Rhizobium* (three OTUs), *Pseudomonas* (three OTUs), *Bacillus* (two OTUs), *Stenotrophomonas* (two OTUs), *Sphingobacterium* (two OTUs), and *Streptomyces* (two OTUs). Four OTUs, OTU430 (*Streptomyces*), OTU41 (*Chitinophaga*), OTU33 (*Sphingobacterium*), and OTU40 (*Cellvibrio*), were significantly correlated with OTU62 in more than one network. The degree (the number of other OTUs that are significantly correlated with an OTU) of OTU62 was higher than average, and the most of the degrees of OTUs correlated with OTU62 were higher than average, too. Thus, OTU62 may play an important role in endophytic networks, even if it does not show significant enrichment in samples with high PCS severity from any of the tissue types.

## Discussion

Endophytes are considered to be the second line of defense against plant disease (Dini-Andreote, 2020), and they vary greatly in different tissue types of plants (Tervet and Hollis, 2002; Schulz and Boyle, 2005; Leo and Dirk, 2008; You, 2008). In this study, we characterized the composition and distribution of endophytic communities in potato tubers, roots, and stems, and traced the source of the endophytic communities. We also examined the effects of PCS on endophytic communities in these three non-lesion tissues.

Endophytes are diverse, and they vary greatly in diversity, composition, and distribution in different plants and different tissue types of plants (Tervet and Hollis, 2002; Schulz and Boyle, 2005; Leo and Dirk, 2008; You, 2008). The bacterial α-diversity and composition of endophytes in potato roots, stems, and tubers had significant differences (*P <* 0.05), and the community diversity of roots was higher than in stems and tubers (Figure 1). This finding is consistent with a dynamic distribution of endophytes where diversity decreases closer to the top of the plant (Rosenblueth and Martínez-Romero, 2004). Potato tubers form later than the roots and, for that reason, the diversity of endophytes in tubers may be lower than that of roots. We found that the bacterial communities followed a gradient distribution from soil to roots to tubers/stems (Figure 2), suggesting that roots may serve as a transmission channel between the soil bacterial community and endophytes in other plant components. The distribution characteristics of endophytes could also be verified by the results of source tracer analysis (Figure 3). Previous studies showed the main source of endophytes to be the surrounding soil environment, air, and seed tubers, which enter into plants through the stomata and damaged areas or where lateral roots appear and then transfer to other plant tissues (Chi et al., 2005; Mano and Morisaki, 2008). In our study, the main source of root endophytes could be traced to the soil, especially in RS, with a small proportion coming from other plant tissues. Stem endophytes could be traced from GS, RS, and root endophytes, and the proportion from these three sources was similar. Tuber endophytes could be traced from GS, RS, RE, and SE (Figure 3). The “unknown” source of stem endophytes accounted for at least half of the proportion, and we speculate that these may be partly derived from the air. No significant differences were observed in the endophyte sources between PCS H and L groups, which was inconsistent with the results of Hu et al. (2020). In their study, soil was the main source of root endophytes from tomato plants infected with bacterial wilt, while endophytes in healthy plants could hardly be traced to the soil (Hu et al., 2020). However, bacterial wilt is a disease that causes wilting of the whole plant, while PCS is characterized by the formation of scab lesions on the surface of the tuber with no obvious symptoms elsewhere. And so, dynamic changes in endophytes may not be significant after disease infection, and the effects on non-lesion tissues of the plant may not be observable. This hypothesis is also consistent with the dissimilarity of the endophytic communities between the H and L groups (Figure 1)—we only found a significant difference between the H and L groups in the soil closest to the potato tuber (GS) and marginally significant differences in tuber endophytes, but no significant differences in rhizosphere soil, stem, or root endophytes.

Endophytes are known to play a positive role in resisting plant disease (Sturz et al., 2000). The structure and diversity of endophytes can change or even recruit specific microorganisms to improve plant resistance to survival stress (Carrión et al., 2019). For example, when bananas and tomatoes were infected with wilt, or potatoes with Blackleg, endophytic diversity in diseased tissues increased (Reiter et al., 2002; Lian et al., 2008; Carrión et al., 2019; Hu et al., 2020). The inoculation of pathogenic *R. solani* into the soil of beets resulted in the recruitment of specific inhibitory strains of *Chitinophagaceae* and *Flavobacteriaceae* to colonize the endophytic community of the roots (Reiter et al., 2002; Lian et al., 2008; Carrión et al., 2019; Hu et al., 2020). *Corynebacterium flavescens* and *Bacillus pumilus* reduced or inhibited the colonization of *Azospirillum brasilense* in roots (Baciliojimenez et al., 2001). In our study, PCS did not cause significant compositional differences in endophytes in non-lesion tissue types—including roots, tubers or stems between high (H) and low (L) infection groups at the community level—but did affect the relative abundance of several specific endophytes. *Delftia* and nitrogen-fixing *Rhizobium* were significantly different between the H and L groups in the stems and tubers. *Delftia* may inhibit various plant pathogens, such as *Xanthomonas oryzae pv. oryzae* and *Rhizoctonia solani* (Han et al., 2005). Cirou et al. (2007) found that several types of *Delftia* bacteria surrounding potato roots could degrade the pathogenic signal molecules of *Pectobacterium atrosepticum*, interfere with the pathogenic signal transduction of pathogenic bacteria, and reduce the occurrence of potato black shin disease. *Rhizobium* may contribute to hormone production, phosphate solubilization, and the suppression of pathogens (Sessitsch et al., 2002). We suspect that *Delftia* and *Rhizobium* play a positive role in PCS control, which we aim to verify in future studies. The pathogen of PCS belongs to the genus *Streptomyces*, and PCS-causing *Streptomyces* are mainly spread via seed and soil. Current control strategies, such as seed potato disinfection and soil fumigation, are attempts to disrupt the seed- and soil-borne pathways of pathogens. The pathogenic strains have been isolated from tuber lesions and GS (Shi et al., 2019), but it is not clear whether pathogens may also occur in non-lesion tissues. Our results showed that the potential pathogen OTU62 could colonize in roots, tubers, and stems. Even with mild or no symptoms of PCS infection, we still detected the pathogen across all tissue types. The 40 OTUs, mainly belonging to *Chryseobacterium*, *Rhizobium*, *Pseudomonas*, *Bacillus*, *Stenotrophomonas*, *Sphingobacterium*, and *Streptomyces*, were significantly correlated with the potential pathogen OTU62. Four OTUs, OTU430 (*Streptomyces*), OTU41 (*Chitinophaga*), OTU33 (*Sphingobacterium*), and OTU40 (*Cellvibrio*), were significantly correlated with OTU62 in more than one network. A multitude of studies have reported that strains of *Chryseobacterium* spp., *Chitinophaga* spp., *Pseudomonas* spp., *Sphingomonas* spp., and *Stenotrophomonas* spp. tend to be enriched in plants in response to different pathogen/pest attacks and promote plant resistance (Liu et al., 2020). The higher network degree of OTU62 in all three endophytic networks further supports the response to PCS across all tissue types, including non-lesion areas. Unfortunately, the potential pathogen OTU62 failed to be isolated in the pure culture experiment. It is worthwhile to verify the pathogenicity of the potential pathogenic *Streptomyces* stains in endophytic communities and gain a greater understanding of their role in the development of PCS in future research.

## Conclusion

Endophyte communities of potato tubers, roots, and stems were significantly different from each other and presented a gradient distribution from soil to root to tuber/stem (GS/RS to RE to TE/SE). Roots are the gateway for the soil bacterial community to enter the plant. About 50% of root endophytes could be traced to the soil community, especially the rhizosphere community, but only about 20% of tuber and stem endophytes could be traced to the soil. PCS significantly reduced Ace and Obs indexes of RE without significantly reducing the indexes of SE and TE. No significant compositional differences to the endophytes were caused by PCS in the non-lesion tissue types, including roots, tubers, or stems, but did affect the relative abundance of several specific endophytes. *Rhizobium* and *Sphingopyxis* were significantly enriched in root endophytes with low PCS severity; *Delftia* and *Ochrobactrum* were significantly enriched in stem endophytes with low PCS severity, and *Pedobacter*, *Delftia*, and *Asticcacaulis* were significantly enriched in tuber endophytes with high PCS severity. The potential PCS pathogen in this study, OTU62, existed in the roots, tubers, and stems even with mild severity of PCS symptoms and held a high network degree in endophytic networks. This study provides novel insight into the composition and distribution characteristics of potato endophytic communities and their response to PCS.

## Data Availability Statement

The datasets presented in this study can be found in online repositories. The names of the repository/repositories and accession number(s) can be found below: NCBI Sequence Read Archive (SRA) under the BioProject number PRJNA657530.

## Author Contributions

ZG and BZ conceived and supervised the project. ZG, BZ, WS, and GS designed the experiment. WS, GS, ML, BW, RL, YY, and TW performed the material preparation, the data collection, and analysis. WS, GS, and ML wrote the first draft of the manuscript. All authors contributed to the study conception, design, commented on previous versions of the manuscript, read, and approved the final manuscript.

## Conflict of Interest

The authors declare that the research was conducted in the absence of any commercial or financial relationships that could be construed as a potential conflict of interest.

## Footnotes

1 https://www.potatonewstoday.com/2020/08/12/global-potato-statistics-an-overview/

2 https://github.com/cozygene/feast

3 https://blast.ncbi.nlm.nih.gov/Blast.cgi

## References

[B1] Arseneault T. Goyer C. Filion M. (2015). *Pseudomonas* fluorescens LBUM223 increases potato yield and reduces common scab symptoms in the field. *Phytopathology* 105 1311–1317. 10.1094/phyto-12-14-0358-r 25961336

[B2] Arslan S. Zakia L. Zhang S. Jing Z. Zechel D. L. Andreas B. (2018). Biological control of potato common scab with rare Isatropolone C compound produced by plant growth promoting streptomyces A1RT. *Front. Microbiol.* 9:1126. 10.3389/fmicb.2018.01126 29899736 PMC5989138

[B3] Baciliojimenez M. Aguilarflores S. Valle M. V. D. Perez A. Zepeda A. Zenteno E. (2001). Endophytic bacteria in rice seeds inhibit early colonization of roots by *Azospirillum brasilense*. *Soil Biol. Biochem.* 33 167–172. 10.1016/s0038-0717(00)00126-7

[B4] Baldani J. Caruso L. Baldani V. L. D. Goi S. R. D?Bereiner J. (1997). Recent advances in BNF with non-legume plants. *Soil Biol. Biochem.* 29 911–922. 10.1016/s0038-0717(96)00218-0

[B5] Berg G. Hallmann J. (2006). “Control of plant pathogenic fungi with bacterial endophytes,” in *Microbial Root Endophytes. Soil Biology*, Vol. 9 eds Schulz B. J. E. Boyle C. J. C. Sieber T. N. (Berlin: Springer), 53–69. 10.1007/3-540-33526-9_4

[B6] Bulgarelli D. Schlaeppi K. Spaepen S. Ver Loren van Themaat E. Schulze-Lefert P. (2013). Structure and functions of the bacterial microbiota of plants. *Annu. Rev. Plant Biol.* 64 807–838. 10.1146/annurev-arplant-050312-120106 23373698

[B7] Cao K. A. L. Costello M. E. Lakis V. A. Bartolo F. Rondeau P. (2016). MixMC: a multivariate statistical framework to gain insight into microbial communities. *PLoS One* 11:e0160169. 10.1371/journal.pone.0160169 27513472 PMC4981383

[B8] Carrión V. J. Perez-Jaramillo J. Cordovez V. Tracanna V. Hollander M. D. Ruiz-Buck D. (2019). Pathogen-induced activation of disease-suppressive functions in the endophytic root microbiome. *Science* 366 606–612. 10.1126/SCIENCE.AAW9285 31672892

[B9] Chen S. Zhang M. Wang J. Lv D. Ma Y. Zhou B. (2017). Biocontrol effects of *Brevibacillus laterosporus* AMCC100017 on potato common scab and its impact on rhizosphere bacterial communities. *Biol. Control* 106 89–98. 10.1016/j.biocontrol.2017.01.005

[B10] Chi F. Shen S. H. Cheng H. P. Jing Y. X. Dazzo F. B. (2005). *Ascending Migration of Endophytic Rhizobia from Roots to Leaves Inside Rice Plants.* Berlin: Springer. 10.1128/AEM.71.11.7271-7278.2005 PMC1287620 16269768

[B11] Cirou A. Diallo S. Kurt C. Latour X. Faure D. (2007). Growth promotion of quorum-quenching bacteria in the rhizosphere of Solanum tuberosum. *Environ. Microbiol.* 9 1511–1522. 10.1111/j.1462-2920.2007.01270.x 17504488

[B12] Compant S. Sessitsch A. Mathieu F. (2012). The 125th anniversary of the first postulation of the soil origin of endophytic bacteria – a tribute to M.L.V. Galippe. *Plant Soil* 356 299–301. 10.1007/s11104-012-1204-9

[B13] Dini-Andreote F. (2020). Endophytes: the second layer of plant defense. *Trends Plant Sci.* 25 319–322. 10.1016/j.tplants.2020.01.007 32191867

[B14] Getahun B. B. (2018). Potato breeding for nitrogen-use efficiency: constraints, achievements, and future prospects. *J. Crop Sci. Biotechnol.* 21 269–281. 10.1007/s12892-018-0014-0

[B15] Hallmann J. Quadt-Hallmann A. Mahaffee W. F. Kloepper J. W. (1997). Bacterial endophytes in agricultural crops. *Can. J. Microbiol.* 43 895–914. 10.1139/m97-131

[B16] Han J. Sun L. Dong X. Cai Z. Sun X. Yang H. (2005). Characterization of a novel plant growth-promoting bacteria strain *Delftia tsuruhatensis* HR4 both as a diazotroph and a potential biocontrol agent against various plant pathogens. *Syst. Appl. Microbiol.* 28 66–76. 10.1016/j.syapm.2004.09.003 15709367

[B17] Hinton D. M. Bacon C. W. (1995). *Enterobacter cloacae* is an endophytic symbiont of corn. *Mycopathologia* 129 117–125. 10.1007/BF01103471 7659140

[B18] Hu Q. Tan L. Gu S. Xiao Y. Xiong X. Zeng W.-A. (2020). Network analysis infers the wilt pathogen invasion associated with non-detrimental bacteria. *NPJ Biofilms Microbiomes* 6 8. 10.1038/s41522-020-0117-2 PMC7021801 32060424

[B19] Ji X. Lu G. Gai Y. Zheng C. Mu Z. (2008). Biological control against bacterial wilt and colonization of mulberry by an endophytic *Bacillus subtilis* strain. *FEMS Microbiol. Ecol.* 65 565–573. 10.1111/j.1574-6941.2008.00543.x 18631174

[B20] Leiminger J. H. Frank M. Wenk C. Poschenrieder G. Schwarzfischer A. (2013). Distribution and characterization of Streptomyces species causing potato common scab in Germany. *Plant Pathol.* 62 611–623. 10.1111/j.1365-3059.2012.02659.x

[B21] Leo V. O. Dirk V. E. J. (2008). Effects of plant genotype and growth stage on the structure of bacterial communities associated with potato (*Solanum tuberosum* L.). *FEMS Microbiol. Ecol.* 64 283–296. 10.1111/j.1574-6941.2008.00469.x 18355298

[B22] Leuchtmann A. (2006). Systematics, distribution, and host specificity of grass endophytes. *Nat. Toxins* 1 150–162. 10.1002/nt.2620010303 1344916

[B23] Lian J. Wang Z. Zhou S. (2008). Response of endophytic bacterial communities in banana tissue culture plantlets to Fusarium wilt pathogen infection. *J. Gen. Appl. Microbiol.* 54 83–92. 10.2323/jgam.54.83 18497482

[B24] Liu H. Brettell L. E. Qiu Z. Singh B. K. (2020). Microbiome-mediated stress resistance in plants. *Trends Plant Sci.* 25 733–743. 10.1016/j.tplants.2020.03.014 32345569

[B25] Lyons P. C. Evans J. J. Bacon C. W. (1990). Effects of the fungal endophyte *Acremonium coenophialum* on nitrogen accumulation and metabolism in tall fescue. *Plant Physiol.* 92 726–732. 10.1104/pp.92.3.726 16667341 PMC1062360

[B26] Ma B. Lv X. Warren A. Gong J. (2013). Shifts in diversity and community structure of endophytic bacteria and archaea across root, stem and leaf tissues in the common reed, Phragmites australis, along a salinity gradient in a marine tidal wetland of northern China. *Antonie Van Leeuwenhoek* 104 759–768. 10.1007/s10482-013-9984-3 23897211

[B27] Mano H. Morisaki H. (2008). Endophytic bacteria in the rice plant. *Microbes Environ.* 23 109–117. 10.1264/jsme2.23.109 21558696

[B28] Meng Q. Hanson L. E. Douches D. S. Hao J. (2013). Managing scab diseases of potato and radish caused by *Streptomyces* spp. using *Bacillus amyloliquefaciens* BAC03 and other biomaterials. *Biol. Control* 67 373–379. 10.1016/j.biocontrol.2013.09.009

[B29] Nejad P. Johnson P. A. (2000). Endophytic bacteria induce growth promotion and wilt disease suppression in oilseed rape and tomato. *Biol. Control* 18 208–215. 10.1006/bcon.2000.0837

[B30] Parks D. H. Tyson G. W. Philip H. Beiko R. G. (2014). STAMP: statistical analysis of taxonomic and functional profiles. *Bioinformatics* 30 3123–3124. 10.1093/bioinformatics/btu494 25061070 PMC4609014

[B31] Reiter B. Pfeifer U. Schwab H. Sessitsch A. (2002). Response of endophytic bacterial communities in potato plants to infection with Erwinia carotovora subsp. atroseptica. *Appl. Environ. Microbiol.* 68 2261–2268. 10.1128/aem.68.5.2261-2268.2002 11976096 PMC127529

[B32] Rosenblueth M. Martínez-Romero E. (2004). Rhizobium etli maize populations and their competitiveness for root colonization. *Arch. Microbiol.* 181 337–344. 10.1007/s00203-004-0661-9 15024554

[B33] Rosenzweig N. Tiedje J. M. Quensen J. F. Meng Q. Hao J. J. (2012). Microbial communities associated with potato common scab-suppressive soil determined by pyrosequencing analyses. *Plant Dis.* 96 718–725. 10.1094/PDIS-07-11-0571 30727523

[B34] Sarwar A. Latif Z. Zhang S. Zhu J. Zechel D. L. Bechthold A. (2018). Biological control of potato common scab with rare Isatropolone C compound produced by plant growth promoting streptomyces A1RT. *Front. Microbiol.* 9:1126. 10.3389/FMICB.2018.01126 29899736 PMC5989138

[B35] Schulz B. Boyle C. (2005). The endophytic continuum. *Mycol. Res.* 109(Pt 6) 661–686. 10.1017/s095375620500273x 16080390

[B36] Sessitsch A. Howieson J. G. Perret X. Antoun H. Martínez-Romero E. (2002). Advances in Rhizobium research. *Crit. Rev. Plant Sci.* 21 323–378.

[B37] Shenhav L. Thompson M. Joseph T. A. Briscoe L. Halperin E. (2019). FEAST: fast expectation-maximization for microbial source tracking. *Nature Methods* 16 1–6. 31182859 10.1038/s41592-019-0431-x PMC8535041

[B38] Shi W. Li M. Wei G. Tian R. Li C. Wang B. (2019). The occurrence of potato common scab correlates with the community composition and function of the geocaulosphere soil microbiome. *Microbiome* 7 1–18. 10.1186/S40168-019-0629-2 30709420 PMC6359780

[B39] Stone Bacon White J. F. (2000). “An overview of endophytic microbes: endophytism defined,” in *Microbial endophytes*, eds Bacon White J. F. (New York, NY: Marcel Dekker), 29–33.

[B40] Sturz A. V. Christie B. R. Nowak J. (2000). Bacterial endophytes: potential role in developing sustainable systems of crop production. *Crit. Rev. Plant Sci.* 19 1–30. 10.1080/07352680091139169

[B41] Sun L. Qiu F. Zhang X. Dai X. Dong X. Song W. (2008). Endophytic bacterial diversity in rice (Oryza sativa L.) roots estimated by 16S rDNA sequence analysis. *Microb. Ecol.* 55 415–424. 10.1007/s00248-007-9287-1 17690836

[B42] Tervet I. W. Hollis J. P. (2002). Bacteria in the storage organs of healthy plants. *Phytopathology* 38 960–967.

[B43] Tomihama T. Nishi Y. Mori K. Shirao T. Iida T. Uzuhashi S. (2016). Rice bran amendment suppresses potato common scab by increasing antagonistic bacterial community levels in the Rhizosphere. *Phytopathology* 106 719–728. 10.1094/PHYTO-12-15-0322-R 27050572

[B44] You J. M. (2008). Dynamic distributes of endophytic fungi from *Camellia sinensis*. *Guihaia* 1 82–85.

